# Srs2: The “Odd-Job Man” in DNA repair

**DOI:** 10.1016/j.dnarep.2010.01.007

**Published:** 2010-03-02

**Authors:** Victoria Marini, Lumir Krejci

**Affiliations:** a Department of Biology, Faculty of Medicine, Masaryk University, Brno CZ-625 00, Czech Republic; b National Centre for Biomolecular Research, Faculty of Science, Masaryk University, Brno CZ-625 00, Czech Republic

**Keywords:** DSB, double-strand break, HR, homologous recombination, NHEJ, non-homologous end joining, PRR, post-replication repair, SDSA, synthesis-dependent single-strand annealing, HU, hydroxyurea, MMS, methyl methanesulfonate, dHJ, double Holliday junction, HJ, Holliday junction, SCE, sister-chromatid exchange, DNA repair, Recombination, Helicases, Srs2

## Abstract

Homologous recombination plays a key role in the maintenance of genome integrity, especially during DNA replication and the repair of double-stranded DNA breaks (DSBs). Just a single un-repaired break can lead to aneuploidy, genetic aberrations or cell death. DSBs are caused by a vast number of both endogenous and exogenous agents including genotoxic chemicals or ionizing radiation, as well as through replication of a damaged template DNA or the replication fork collapse. It is essential for cell survival to recognise and process DSBs as well as other toxic intermediates and launch most appropriate repair mechanism. Many helicases have been implicated to play role in these processes, however their detail roles, specificities and co-operativity in the complex protein-protein interaction networks remain unclear. In this review we summarize the current knowledge about Saccharomyces cerevisiae helicase Srs2 and its effect on multiple DNA metabolic processes that generally affect genome stability. It would appear that Srs2 functions as an “Odd-Job Man” in these processes to make sure that the jobs proceed when and where they are needed.

## 1 Introduction

The genome is constantly threatened by various damaging agents and maintaining its integrity is crucial in all organisms. Several repair pathways have been implicated in the removal of different types of lesions from DNA. Among them, homologous recombination (HR) plays a key role in repair of double-strand breaks (DSBs). Although HR is a highly important repair mechanism, it has to be regulated to prevent it from interfering with other DNA repair pathways, generating toxic intermediates, or blocking the progression of the replication fork. Therefore, it is not surprising that cells have evolved mechanisms that counteract untimely HR events. In the yeast *Saccharomyces cerevisiae*, one of the pathways responsible for regulation of HR requires the action of the *SRS2* gene product. Mutations in the *SRS2* gene exhibit pleiotropic recombination phenotypes ranging from anti-recombinogenic in one aspect to pro-recombinogenic in another. In addition to its role in HR, Srs2 is also involved in other DNA metabolism processes, including post-replication repair (PRR), preservation of replication fork integrity, DNA-damage checkpoint responses, DNA triplet maintenance and non-homologous end joining (NHEJ).

## 2 Biochemical properties

**Fig. 1:**
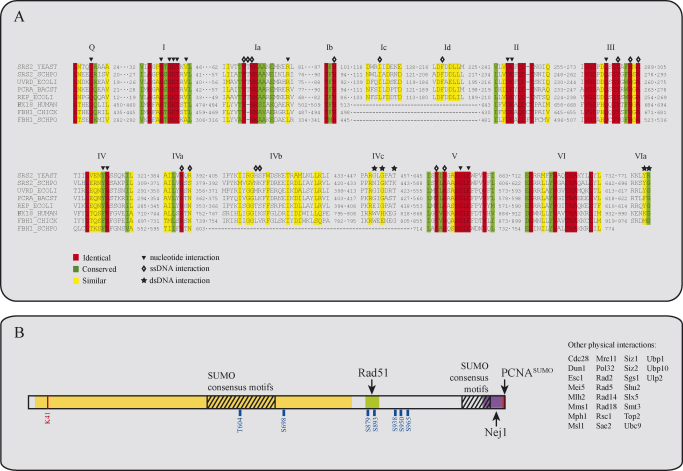
Homology of Srs2 with other known helicases. (A) Several members of the SF-I family helicases and their alignment to Srs2 using CLUSTALW. Helicase motifs are indicated above the sequences. Color-coding is based on amino acid conservation. Symbols indicate important interaction amino acids within UvrD. (B) Schematic representation of Srs2. The helicase domain is colored in yellow, the Rad51 interaction domain in green and the PCNA^SUMO^ interaction domain in red. Striped areas contain SUMO consensus motifs. The marked amino acids in blue are phosphorylation sites. K41 represents the Walker type A motif.

The *SRS2* gene encodes a superfamily I DNA helicase with homologies to the bacterial helicases Rep, PcrA and UvrD [1–3] (Fig. 1). In contrast to these prokaryotic helicases, Srs2 contains an additional C-terminal region that mediates many protein–protein interactions and is also a target for post-translational modification. Interestingly, a previously performed large scale 2-hybrid screen using Srs2 as bait identified 166 potential interacting proteins and some of which are shown in Fig. 1 [4]. Biochemically, Srs2 possesses strong ssDNA-dependent ATPase activity with a kcat ≥3000 min^−1^ unwinds DNA with 3–5′ polarity [5,6], and the Walker A motif is absolutely required for both ATPase and helicase activities [7]. DNA with a 3′ overhang containing at least 10 nucleotides is the preferred substrate for its helicase activity. Srs2 is also able to unwind substrates containing forks, flaps, D-loops as well as 5′ ssDNA overhangs and blunt end dsDNA substrates ([6]; Marini and Krejci, unpublished data). Srs2 is also able to unwind *in vitro* structures that resemble D-loops recombination intermediates and this activity is stimulated by Rad51 bound to dsDNA [8]. However, recent experiments have shown that the helicase Mph1 is more efficient than Srs2 in dissociating D-loops formed by Rad51 [9]. In addition, the single-strand DNA binding proteins, RPA or SSB, enhance Srs2 unwinding of long substrates, by preventing the reannealing of the separated strands [6]. Srs2 is able to translocate on ssDNA as a monomer, with an average processivity of almost 1600 nt with a rate of 300 nt/s, as revealed by analytical centrifugation [10]. Interestingly, it has been proposed for other helicases that translocase and helicase activities are separate functions and oligomerization might be required for the latter [11]. Further mechanistic analysis of the helicase and translocase activities of Srs2, investigation of the roles of Srs2 interacting partners, and structural characterization of Srs2 will help to understand its biological roles.

## 3 Anti-recombinase activity

HR contributes to genomic integrity and the repair of DSBs as well as acting during the recovery of damaged replication forks. However, HR must be also tightly regulated to prevent untimely events that could interfere with other DNA repair mechanisms as well as during replication fork progression. It has been noted that damaged DNA, blocked replication forks, or nucleoprotein complexes generated by the HR machinery can trigger cell cycle arrest and even cause cell death in certain genetic backgrounds [12–15]. Several pathways are involved in the elimination of undesirable HR intermediates. Interestingly, they all include the action of a helicase but use different mechanisms and act at different stages. One pathway involves the activity of the Srs2 helicase, which allegedly suppresses HR events at an early stage by dismantling the Rad51-presynaptic filament. A second mechanism involves the action of Mph1 or human RTEL that have been suggested to influence the efficiency of HR by disrupting the D-loop intermediate produced by the Rad51 recombinase [9,16]. Finally, BLM/Sgs1 together with Top3 is required for dissolution of dHJ or other recombination intermediates [17–19]. Here, we will describe in detail the anti-recombinase activity of Srs2 protein. The role of other helicases during HR will be the focus of other accompanying reviews.

Genetic studies were the first to suggest a possible role for Srs2 as an anti-recombinase. Mutations in the *SRS2* gene lead to a hyper-recombination phenotype due to inappropriate channeling of the lesions into the homologous recombination pathway [20–23]. The need for appropriate regulation of HR is most clearly illustrated by the near lethal phenotype of the *srs2 sgs1* double mutant. Deletion of recombination genes can efficiently suppress the severe phenotype of this double mutant as well as the *srs2* mutant [12,20,24–26]. These genetic data suggest that *sgs1 srs2* cells accumulate toxic recombination intermediates that cannot be resolved in the absence of the Srs2 and Sgs1 helicases.

To directly demonstrate the effect of Srs2 on the efficiency of recombination, biochemical studies have shown that catalytic amounts of Srs2 protein can dramatically inhibit the formation of Rad51-mediated joint molecules and D-loop formation [27,28]. Additional biochemical and electron microscopy analysis revealed that Srs2 efficiently dismantles the presynaptic filament formed by Rad51, an early HR intermediate. This is further enhanced in the presence of RPA that prevents re-nucleation of Rad51 on cleared ssDNA [27,28]. The helicase activity does not appear to be responsible for the dissociation of these intermediate molecules due to its modest processivity as well as lower activities on these structures [6,9,27]. Rather, ATP hydrolysis-fueled translocase activity is necessary for Srs2 to dismantle Rad51 filaments; mutants that cannot bind or hydrolyze ATP fail to disrupt Rad51-presynaptic filaments [7]. In agreement with the biochemical data, these ATPase dead mutants also show similar sensitivities to genotoxic agents, a hyper-recombination phenotype and synthetic interactions when compared to the *srs2* deletion mutant [7]. It has been suggested that Srs2 activity might be guided to the Rad51-filament via its direct physical interaction with Rad51 [27]. In agreement with this view, mutants of Rad51 that fail to interact with Srs2 are resistant to its anti-recombinase activity [29]. Accordingly, a mutant version of Srs2 that retains wild type levels of ATPase and helicase activities, but fails to interact with Rad51 is compromised for its anti-recombinase function [30]. The mechanism by which Srs2 dismantles Rad51 seems to be the result of ATP-driven motor activities of Srs2 that are capable of dissociating not only DNA structures but also protein–DNA complexes. Recent evidence suggests that the direct interaction between Srs2 and Rad51 is used both to target Srs2 to HR intermediates and to trigger ATP hydrolysis within the Rad51 filament, causing Rad51 to dissociate from DNA [10]. In line with this, the crystal structures of Rad51 and RecA reveal the importance of adjacent monomers in the coordination of ATP binding [31–33]. It is therefore intriguing to hypothesize that Rad51 filaments could actually sense/coordinate the Srs2 translocase activity through its own ATP hydrolysis-mediated affinity towards ssDNA, thus allowing more efficient clearing of the nucleoprotein filament by Srs2.

## 4 Role of Srs2 in replication fork maintenance and post-replication repair (PRR)

During replication, a damaged DNA template frequently blocks the progression of the replication fork. Unless stalled forks are able to restart, the cells cannot complete replication, which leads to cell cycle arrest and cell death. Several checkpoint-related proteins have been implicated in reducing the frequency of HR, that otherwise could lead to destabilization of replication forks and generation of toxic HR intermediates [34,35]. Srs2 is directly implicated in the replication checkpoint. This is based on the fact that *srs2* mutants fail to fully activate a Rad53-dependent DNA damage response, which is required to slow DNA replication [36]. Srs2 is also required for recovery and adaptation in response to checkpoint-mediated cell cycle arrest [14,36]. It still needs to be addressed whether the role for Srs2 during DNA-damage checkpoint activation requires its anti-recombinase activity or if this is dependent on its role in determining the fate of the replication fork.

**Fig. 2:**
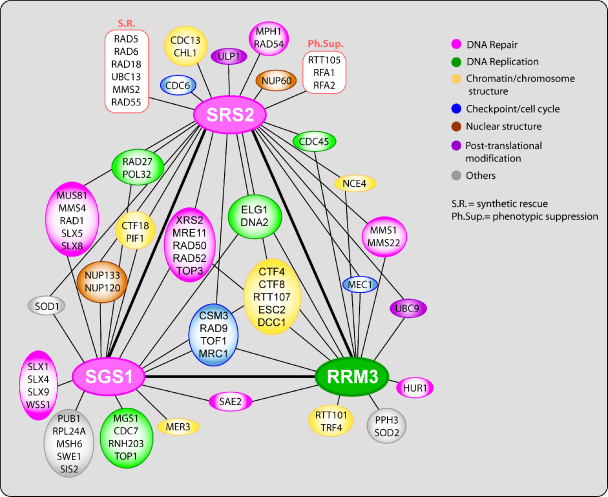
Complexity of genetic interactions between *SRS2*, *SGS1*, and *RRM3* helicases as well as other genes as a part of a genome integrity network.

Several mechanisms have been described that would provide multiple options for cells to counteract damage while replicating their DNA. Mechanisms that control recombination and other DNA repair processes during replication are again distinguished by the action of several helicases. Among them, *SRS2*, *SGS1* and *RRM3* genes show overlapping roles in genome maintenance as demonstrated by growth impairments or lethal phenotypes when mutations in these genes are combined [22,37–41]. It has been established that these helicases perform specific functions that cannot be executed by any another helicase (Fig. 2). As mentioned above, the inhibition of these alternative pathways in Srs2 mutants leads to synthetic lethal phenotypes, which can be suppressed by preventing recombination. This suggests that the helicase double mutants accumulate toxic HR intermediates, further supporting the need for tight regulation of recombination during DNA replication [22,39]. Rrm3, together with other components of the replication fork machinery ensures fork progression in the presence of replication blockages and prevents extended fork pausing that may occasionally lead to fork collapse [39,42–44]. The Sgs1 helicase seems to promote fork restart using HR via generation of recombination intermediates, as well as mediating their resolution [22]. The second mechanism involved in the restart of collapsed replication forks requires the action of the Srs2 helicase and other members of the *RAD6* epistasis group, which are required for PRR. This pathway may also lead to the formation of repair intermediates that require processing by the activity of the Sgs1 helicase [45]. Alternatively, another yeast helicase Mph1 has been implicated in an error-free DNA damage bypass pathway that requires genes from HR [46].

The *SRS2* gene was first identified as a suppressor of DNA damage sensitivity of both *rad6* and *rad18* mutants (*S*uppressor of *R*AD *S*ix mutant *2*) and this suppression requires functional HR [3,47–49]. Accordingly, Srs2 is also actively recruited to replication forks via its interaction with SUMOylated PCNA and this recruitment favors the use of PRR [50–52]. At the replication fork, Srs2 acts as a molecular switch between PRR and HR by channeling DNA lesions into the *RAD6* pathway while preventing the use of an alternative recombination repair [21,26,53]. Little is known about the molecular details of PRR, but genetic studies have revealed an intricate system of several independent pathways, comprising error-free and error-prone sub-pathways that allow the bypass of replication-blocking lesions [54]. It is possible that Srs2 could act at several stages of PRR, involving both early as well as late roles. It could prevent substrates assigned for PRR to be repaired by recombinational mechanisms by directly disrupting Rad51-filaments. It could also prevent or reverse Rad51 binding to repair intermediates, which could be important for preventing the nascent strand from pairing to generate chicken-foot structures that occur at stalled replication forks [55]. It is also possible that Srs2 could facilitate the generation of substrates needed for the error-prone or error free sub-pathways of PRR [45,54]. Such ability would help to explain the increased UV-sensitivity of *rad6/rad18* strain in the *srs2* mutant [23,56]. It has been noted that Srs2 is required for some or all branches of PRR where it could play an important role in the decision between error-free or error-prone PRR pathways [57–59]. However, further studies are needed to elucidate the mechanistic role of Srs2 in PPR pathways.

## 5 Srs2 as part of a recombination “quality control” mechanism

Recently, the cellular role of Srs2 at replication forks and in the regulation of HR was addressed. A decreased requirement for Rad52 activity during Rad51 focus formation in the *srs2*Δ mutant was observed, suggesting that Srs2 antagonizes Rad52 in the formation of Rad51 filaments [60]. This idea was further supported, by the ability of Rad52 protein to suppress the inhibitory effect of Srs2 on Rad51-mediated strand exchange [60]. Furthermore, Rad51 mutants, originally isolated as Rad52-interaction deficient mutants [61], also appeared defective for the interaction with Srs2, suggesting that these proteins compete for the same interaction region on Rad51 [29]. As expected, these Rad51 mutants show resistance to the action of Srs2 as well as an inability of Rad52 to overcome the RPA inhibition during Rad51-mediated strand exchange [29]. Accordingly, an Srs2 mutant protein that fails to interact with Rad51 is compromised for anti-recombinase function *in vitro* as well as *in vivo* [10,30]. However, additional mechanisms could still exist that restrict the recruitment of HR proteins to replication forks even in the absence of Srs2.

**Fig. 3:**
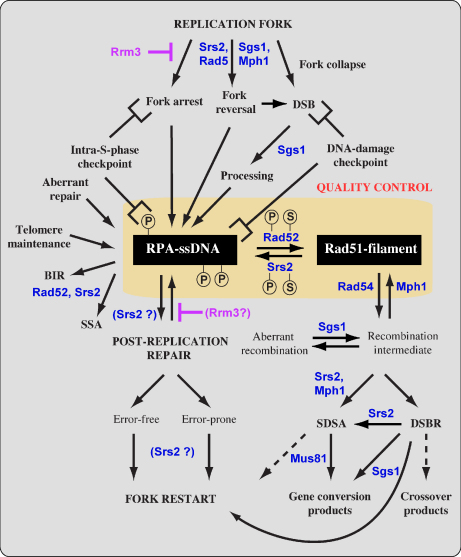
Multifunctional role of Srs2 and other helicases during DNA repair and homologous recombination. RRM3 prevents the replication fork from stalling and collapsing. However, when this happens stalled or broken forks activate the intra-S-phase or DNA damage checkpoints, respectively. Also the RPA–ssDNA complex triggers a checkpoint response. Srs2 and Sgs1 serve distinct as well as overlapping functions in the regulation of recombination bypass of replication forks. Collapsed or broken forks are channeled either to Rad51-mediated recombination or Rad51-independent PRR. Sgs1 is involved in processing DSBs to generate 3′ tails for Rad51 filament formation and together with other helicases facilitate fork reversal. Srs2 and Rad52 are part of a “quality control” mechanism that influences the efficiency of repair via alternative routes. The quality control mechanism allows the cell to regulate the outcome of the intermediate, RPA–ssDNA or Rad51-filament, formed during repair. Srs2 and Mph1 or Sgs1 also play a downstream role in SDSA and DSBR, respectively. A synthetic lethal interaction between these helicases is due to the generation of toxic recombination intermediates, as deletion of recombination genes suppresses the lethality. The only difference is that deletion of the *RAD52* gene that does not alleviate the synthetic phenotype, arguing for a role of a Rad52-mediated and Rad51-independent process (BIR or SSA) as an alternative repair pathways. However, it should also be noted that Rad51 could be required for a specific role at blocked forks as suggested for bacterial RecA. It was proposed that RecA binds to the ssDNA region on the blocked lagging strand, and that this RecA filament invades the homologous leading strand, thus forming a reversed fork [103,104]. Also in yeast, recombination and template switching had previously been considered as separate mechanisms. Recent studies suggest that Rad51 and Rad18 have overlapping functions in the formation of sister-chromatid joint molecules after MMS treatment [45].

Based on our current knowledge, a model for a recombination “quality control” mechanism for the repair of DSBs and damaged replication forks can be proposed (Fig. 3). In one pathway of this model, Srs2 scans the chromosomes for inappropriate Rad51-filaments. This is in agreement with recent observations showing that deletion of *SRS2* results in an increase in the number of Rad51 and Rad54 foci even at genomic locations that are not relevant to HR, confirming the loading of HR proteins at inappropriate sites [60]. Srs2 anti-recombinase activity is able to remove Rad51 molecules from ssDNA that are subsequently occupied by RPA, thus making the DNA inaccessible for Rad51 reloading. In addition, the RPA-coated ssDNA, as a common structure generated at the sites of DNA damage or as a result of Srs2 anti-recombinase activity, is a target for modification as well as responsible for recruitment of the checkpoint machinery [15,62,63] and/or Rad18 [64]. The damage checkpoint once recruited may participate in the choice of repair pathways. If required, Srs2 could also channel substrates derived from other repair processes into the PRR pathway. When the cell favors HR, the second pathway of this model (Fig. 3) involving Rad52 mediator activity will antagonize Srs2 by nucleating Rad51-filaments [65–67]. Therefore, an equilibrium between these pathways might exist allowing cells to quickly and appropriately react to the incoming signal. Perhaps post-translational modifications or additional factors could shift this equilibrium accordingly as a part of a quality control mechanism. Future studies should provide details of how such regulation determines the fate of DNA repair at replication forks. Interestingly, the C-terminal region of Srs2 can be envisioned as a control node that integrates motor activity of Srs2 with protein interactions (Fig. 1). Similarly, Rad52 is also modified by phosphorylation and SUMOylation [68,69]. In summary, it is attractive to speculate that a cooperative or coordinated regulatory mechanism exists that determines the outcome of repair events at common HR intermediates.

## 6 Srs2 and additional roles in DNA repair

Interestingly, even though Srs2 suppresses spontaneous recombination events via its anti-recombinase activity, the same activity also facilitates DSB repair by the synthesis-dependent single-strand annealing (SDSA) pathway [70,71]. Srs2 channels recombination intermediates into non-crossover pathways to minimize crossing over. During SDSA, Srs2 could facilitate strand displacement and/or displacement of Rad51 from non-invading ssDNA in the D-loop structure, thus preventing second-end capture. A role of Srs2 downstream of the strand invasion step and possibly in promoting double-strand break repair is further supported by the failure to detect accumulation of recombination intermediates in *srs2* or *pol30* mutants in contrast to *sgs1* and *ubc9* mutants [72,73]. In addition, the Srs2 mutants defective in ATP binding and hydrolysis display a hyper-recombination phenotype that is even more pronounced than in a *srs2* deletion strain, suggesting that when Srs2 is absent, recombination is used to repair spontaneous DNA damage, but when a defective Srs2 protein is present, it might impede alternative pathways of repair [7]. The ability of Srs2 to remove Rad51 from ssDNA might also be required during other alternative repair pathways, including break-induced replication (BIR) and single-strand annealing (SSA) [74–77]. It has been recently observed that Srs2 is recruited to DSBs by Nej1 to promote NHEJ via SSA-like repair [75]. In this pathway, Rad52-dependent strand annealing and subsequent repair are probably achieved by Srs2-mediated removal of Rad51 from ssDNA overhangs. This mechanism could help to explain why Srs2 is recruited to HR foci independent of Rad51 [60].

Srs2 has also been implicated in maintaining the stability of trinucleotide repeats, which are known to be responsible for a series of hereditary neurological disorders. Mutation in *srs2* leads to triplet repeat expansion. The function of Srs2 in preventing expansion is dependent on its helicase activity, requires connection with polymerase δ, but it is not dependent on recombination [78]. Srs2 is more efficient than Sgs1 and UvrD in unwinding substrates that mimic the triplet repeat hairpin, which is believed to be the relevant intermediate during expansions *in vivo* [79,80]. However, a recent study of long trinucleotide repeats established dependency, but to different degrees, on *RAD52* and *RAD51*, in both *srs2* and *sgs1* mutants [81]. This discrepancy could reflect a possible repeat size threshold that triggers HR [81]. In general, Srs2 seems to be responsible for prevention of repeat expansions that could arise via hairpin formation on the nascent lagging strand. In contrast, mutation in *SGS1* leads to contractions, suggestive of a role in unwinding hairpins that form on the lagging-strand template [81]. Nevertheless, other data point towards a role for Sgs1 in large-scale repeat expansions, possibly via a template switching mechanism [82].

## 7 Regulation of Srs2 activities

The roles of Srs2 in a number of different repair processes necessitate the proper regulation of its activity. Regulation and communication of Srs2 with cell cycle checkpoint pathways may provide a means to chose the most appropriate method to overcome stalled replication forks and other types of DNA damage. The post-translational modification of Srs2 could regulate the availability of specific DNA substrates that either favor homologous recombination or promote the use of alternate pathway to bypass the damage, such as template switching. In general, *SRS2* is expressed at low levels and its expression is coordinated with DNA synthesis genes, which are induced at the G1-S transition. In addition, Srs2 expression is induced by UV irradiation, but this is restricted to G2 cells [83].

Srs2 is also regulated by phosphorylation, which occurs following activation of the intra-S-phase checkpoint and this requires Dun1, Mec1 and Rad53 kinases [36]. Several studies have suggested that regulation of Srs2 can also be achieved through its recruitment to replication forks via its interaction with SUMOylated PCNA [50,51]. Accordingly, Srs2 was shown to form S-phase foci in a PCNA-SUMO dependent fashion; mutants deficient for PCNA SUMOylation or Srs2 lacking the SUMO interaction domain failed to form replication foci [60]. Conversely, recruitment of Srs2 to DSB foci is independent of PCNA interaction and also does not require interaction with Rad51. Instead, SUMOylation of Srs2 and the protein Siz1 plays a role in Srs2 recruitment to these foci [60]. Thus, recruitment of Srs2 to replication forks and other sites of recombination are genetically distinct processes. Perhaps SUMOylation of Srs2 itself is involved in its recruitment, as Srs2 was shown to interact with Smt3 (SUMO) and is SUMOylated both *in vitro* and *in vivo* ([84,85]; Krejci and Zhao, unpublished data). It is also possible that Srs2 regulation is under the direct control of components of the PRR pathway. For example, ubiquitination of Srs2 mediated by either Rad6 or Mms2/Ubc13 ubiquitin-conjugating activities could regulate its activities or recruitment. Understanding the nature of Srs2 regulation will help to clarify the complex relationships between DNA replication, repair and recombination and will be a key challenge for the future.

## 8 The search for human homologs

Srs2 shares a core architecture with other members of the SF-1 family of helicases, including UvrD, PcrA, and Rep [86–88]. UvrD appears to be both structural and functional homolog of Srs2 as it also disrupts formation of RecA filaments and has a role in replication fork maintenance [89,90]. Importantly, Srs2 also contains an additional C-terminal region that possesses many interaction as well as regulatory domains that may be responsible for its other specialized functions (Fig. 1). Fission yeast contains a sequence homolog of Srs2 that shares a number of phenotypes with its budding yeast Srs2 homolog, including hyper-sensitivity to DNA damaging agents, hyper-recombination, and several synthetic lethal interactions [91,92]. However, unlike in budding yeast, the fission yeast homolog is not required for channeling lesions to the PRR pathway [92,93] and can act on stalled or collapsed replication forks independently of PRR [93]. Interestingly, *S*. *pombe* (unlike *S*. *cerevisiae*) contains an additional Srs2-like protein, the F-box helicase Fbh1 that appears to serve in an overlapping manner with Srs2 to alleviate stress caused by DNA damage [94,95]. Mutation in the fission yeast *FBH1* gene leads to a phenotype and genetic interactions that resemble those of *srs2* mutant, suggesting that spFbh1 does indeed act as an anti-recombinase [95–97]. In addition, the expression of hFBH1 can partly complement the *srs2* phenotypes in *S*. *cerevisiae* [98] and mutation of chicken *FBH1* in DT40 cells leads to a slight increase in SCE [94]. Despite their functional similarities, spFbh1 and spSrs2 likely target different HR intermediates [95]. In contrast to Srs2, spFbh1 does not seem to interact with Rad51 and the deletion mutant does not affect spontaneous recombination at direct repeats [95]. The duplication of the *SRS2* locus in certain species suggests that gene duplication might have facilitated their rapid evolution towards specialized functions [99]. It also remains possible that some of the RecQ helicases also possess one or more of the activities of the Srs2 protein (see accompanying reviews). For example, similar to Srs2, RecQL5 was shown to displace Rad51 from ssDNA and acts to suppress recombination in human cells [100]. In addition, BLM and RTEL1 were also shown to inhibit Rad51-mediated SDSA reactions by dissociating D-loops [16,101], this however might reflect an alternative pathway, similar to yeast Mph1 [9].

It is apparent that cells possess a number of over-lapping and compensating anti- and pro-recombination activities associated with Srs2, Fbh1, RecQ helicases, and other helicases (FML1, RTEL1 and others). Each of these helicases contributes to genome stability without strict functional conservation among species. It is likely that the distinct roles played by these helicases are a direct consequence of their association with other cofactors. However, functional redundancy clearly exists between this group of helicases, which illustrates the importance of regulating recombination reactions. Despite the apparent functional overlap, mutants in these helicases clear exhibit distinct cellular phenotypes as well as characteristics indicative of specific functions alone or in combination with other factors. Understanding how these activities cooperate and compensation for each other will continue to provide valuable insights for understanding genome maintenance.

## Conflict of Interest

None.
