# Corrigendum to “Srs2: The ‘Odd-Job Man”’ [DNA Repair 9 (2010) 268–275]

**DOI:** 10.1016/j.dnarep.2012.04.002

**Published:** 2012-06-01

**Authors:** Victoria Marini, Lumir Krejci

**Affiliations:** a Department of Biology, Faculty of Medicine, Masaryk University, Brno CZ-625 00, Czech Republic; b National Centre for Biomolecular Research, Faculty of Science, Masaryk University, Brno CZ-625 00, Czech Republic

The author wishes to inform the readers that there is a correction to the Acknowledgment section. The correct Acknowledgment section is as follows:

The preparation of this article was supported by Wellcome International Senior Research Fellowship (WT076476), Czech Science Foundation grants (GACR 301/09/1917 and GACR 203/09/H046) and Ministry of Education, Youth, and Physical Training of the Czech Republic grants (MSM0021622413 and LC06030).

